# Effects of Drinking Water Quality Parameters on Egyptian Cattle Farm Performance Indicators

**DOI:** 10.1002/vms3.70261

**Published:** 2025-02-21

**Authors:** Mohammed A. Kamal, Mahmoud A. Khalaf, Zakia A. M. Ahmed, Jakeen ELjakee, Hossam Mahmoud, Rashed A. Alhotan, Elsayed Osman Hussein, Branislav Galik, Ahmed Ali Saleh

**Affiliations:** ^1^ Department of Veterinary Hygiene and Management, Faculty of Veterinary Medicine Cairo University Giza Egypt; ^2^ Department of Microbiology, Faculty of Veterinary Medicine Cairo University Giza Egypt; ^3^ Department of Animal Production, College of Food & Agriculture Sciences King Saud University Riyadh Saudi Arabia; ^4^ Al Khumasia For Feed and Animal Products Riyadh—Olaya—Al Aqareyah Riyadh Saudi Arabia; ^5^ Institute of Nutrition and Genomics Slovak University of Agriculture in Nitra Nitra Slovakia; ^6^ Department of Poultry Production, Faculty of Agriculture Kafrelsheikh University Kafr El‐Sheikh Egypt

**Keywords:** beef, dairy, housing, hygienic risk factors

## Abstract

In both beef and dairy farming, the water quality (WQ) is of utmost importance, as it can significantly influence various cattle performance indicators (PIs). This study, conducted in Egyptian cattle farms experiencing emerging epidemics, aimed to scrutinize the impact of WQ on PIs. A comprehensive survey, involving 132 farms, was carried out using a questionnaire to identify hygiene‐related risk factors (HRFs) that affect PIs. In parallel, 132 water samples were meticulously collected, subjected to analysis, and statistically evaluated to establish correlations between WQ parameters and PIs. Depending on the studied parameter (pH, total dissolved solids [TDS], hardness, chloride, nitrate, sulphate, total colony count [TCC] and total coliform count [TCFC]), the permissible limits were exceeded in a notable percentage of the water samples (from 13% to 86.3%). These parameters showed a significant correlation (*ρ* = 0.30–0.64) with feed conversion ratio (FCR) in the case of beef farming, the lowest being for pH (*ρ* = 0.23). Similarly, significant correlations (*ρ* = 0.34–0.69) were found with dairy‐fed efficiency, apart from pH, which showed no correlation (*ρ* = 0). Furthermore, specific WQ parameters statistically emerged as predictors for different PIs. High nitrate was the most influential predictor across all beef and dairy PIs, followed by TDS, hardness, sulphate and microbial count. HRFs such as housing system, bedding type, water source, water tank and pipe type, drinker lining, herd size and cattle breed, demonstrated weak to moderate significant correlation with PIs. To conclude, WQ exerts a considerable impact on cattle PIs with the potential influence of on‐farm HRFs. As a result, it is imperative to consider WQ when formulating rations, implementing alternative hygienic practices, and selecting appropriate water treatment methods for cattle farming.

## Graphical abstract

Drinking water quality significantly impacts beef and dairy performance indicators, with notable correlations among various water chemical parameters, including TDS, hardness, chloride, sulphate and nitrate levels. There are correlations in the levels of total coliform count and total coliform count between each other. Seasonal variations in water microbial counts were observed, and other risk factors were found to influence certain performance indicators. In addition, more studies are required to explore waterborne microbes and biofilm formation in water sources used for cattle.

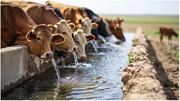

## 1 Introduction

Water, an essential nutrient, ranks second only to oxygen in supporting life and optimizing bovine growth, lactation and reproduction. Bovines have higher water requirements per unit of body mass than any other mammal (Kamal et al. 2023). A significant portion, ranging from 70% to 97%, of cattle's water intake comes from drinking water. Furthermore, the quality of the consumed water plays a critical role, exerting an impact on cattle health and productivity. Water quality (WQ) depends on factors that can act upon the water source. Along this route, the water can suffer abiotic and biotic contamination, which may involve dissolved nutrients or direct urine and faeces contamination (Kamal et al. 2019).

The assessment of drinking WQ predominantly revolves around key factors. Drinking WQ implies physical examination (colour, odour, turbidity, temperature), chemical analysis (pH, hardness, total dissolved solids [TDS], chloride, nitrates and sulphates) and microbiological testing (commonly for total colony count [TCC], total coliform count [TCFC], faecal coliform and tests for specific pathogens). Exceeding the permissible limits of these parameters is particularly detrimental to drinking WQ (Jensen and Vestergaard 2021).

Detrimental limits of drinking WQ parameters impact cattle herd performance indicators (PIs). In beef production, this includes PIs like feed conversion rate (FCR), dry matter intake (DMI), weight gain (WG) and fattening period (FP) (Phillips et al. 2015). In dairy farming, PIs encompass fed efficiency (FE), DMI and daily milk yield (DMY) (Alves et al. 2017). In addition, seasonal climate factors and certain farm‐related risk factors such as operation type, cattle breed, housing conditions and water distribution systems also influence cattle PIs (Abdelhafiz et al. 2021; Zhang et al. 2022; Saleh et al. 2023).

The study's objective was to examine dairy and beef farms in Egypt, evaluating the implications of drinking WQ on the health and performance of cattle. It aimed to determine whether a correlation exists between selected PIs in beef and dairy cattle and the presence of specific contaminants in the water they consume, which are known to cause significant health and performance issues.

## 2 Methods

### 2.1 Field Survey

#### 2.1.1 Study Area

A field investigation was carried out across four regions across Egypt: West Delta (Behira, Alexandria desert road), Middle Delta (Menoufia, Gharbia), East Delta (Kaluobia, Sharkia, Dakahlia, Ismailia desert road) and Upper Egypt (Fayoum, Beni‐Suef, Minya). Water of 1‐L samples were collected from representative water troughs according to troughs count within each adult animal housing at a total of 132 farms situated in the surveyed areas, including beef (60 farms), dairy (60 farms) and dairy‐beef mixed (12 farms).

#### 2.1.2 Study Design

The study protocol involved specific steps designed to assess the hygienic quality of the water consumed by cattle on the farms. To achieve this goal, water samples were collected for a chemical examination and the indicator microbes were counted. The farms were selected based on their history of cattle health issues related to drinking water in the surveyed region. A structured questionnaire was formulated to demonstrate the hygienic risk factors present within each farm. The collected data was then analysed to identify the factors associated with issues in beef and dairy PIs.

#### 2.1.3 Questionnaire Survey

The questionnaire contained comprehensive farm identification information and details regarding hygienic risk factors. These factors encompassed various aspects such as housing characteristics (e.g., housing type, contact with other animals' species, waste handling, carcass disposal and bedding type), as well as water‐related attributes (e.g., water source type, tank type, pipe type and drinker lining). In addition, the questionnaire recorded cattle PIs.

For beef performance, PIs were DMI, final body weight (FBW), FCR, WG, FP, initial age (IG) and initial weight (IW), following the criteria set by El Emam and El Jalii (2010). The dairy PIs were DMI, DMY, and FE, calculated as milk yield divided by DMI, following the guidelines provided by Manzanilla‐Pech et al. (2016). All the data were collected from clinical records of the farm and through interviews with farm owners and veterinarians.

#### 2.1.4 Cattle Farms Descriptions

In many of the surveyed dairy and large beef farms, the predominant housing type was loose/free stalls. In this setup, animals are grouped and housed in separate yards, each equipped with a manger and water trough situated beneath sheds. These yards offer a free space of approximately 7–10 m^2^ per animal. Notably, most of these yards lack a proper drainage system, leading to the accumulation of manure. There was only one exception, a closed farm that houses cows in cubicles/free stalls. Water is consistently accessible in these farms, sourced from the public network, surface water or underground pumps. This water serves various purposes, including drinking, washing and maintaining milking hygiene.

The majority of the small beef and smallholder dairy cattle farms consisted of individually owned cow sheds situated in various provinces, following a traditional design commonly seen in rural Egypt. These cow sheds are referred to as tie‐stalls. They were typically characterized by rudimentary constructions using block bricks, featuring wooden doors and having windows on both sides of the shed. The ceilings were primarily constructed from wooden bars covered with straw, and during the winter season, they were often covered with plastic sheets. The flooring in these sheds is composed of dirty soil, and the removal of manure is typically carried out manually and irregularly. Water is primarily supplied via tap water, which is often chlorinated.

In some farms, routine flushing protocols for water troughs were observed, where troughs were flushed daily or weekly to maintain hygiene. However, other farms lacked clear protocols for flushing, leading to potential water contamination issues.

#### 2.1.5 Water Sampling

A total of 132 water samples were collected from three different source types: ground, surface and commercial tap water. These water samples were evenly collected first time in winter (December, January, February) and the second time in summer (June, July, August) seasons from all the farms included in the survey.

The water samples were carefully collected in separate clean and dry 1 L plastic bottles with screw caps for chemical examination. For the microbiological analysis, we used clean and dry 1 L glass bottles with screw caps which were sterilized in a hot air oven at 170°C for 60 min. These glass containers were rinsed multiple times with sample water before collecting the samples. All samples were stored at 4°C and analysed within 48 h of collection.

Simultaneously, water samples were directly collected using a Dip‐Slides method (Liofilchem), specifically employing CONTACT SLIDE CHROM 2 (ChromaticTM Coli Coliform/Plate Count Agar + TTC + Neutralizing) Flex Dip‐slides. This method utilizes a chromogenic selective medium for detecting and counting *Escherichia coli* and coliforms, along with a non‐selective medium for determining the total bacterial count, following the guidelines outlined in ISO 18593:2004 (2004).

Each sample was carefully labelled and tagged to indicate its source, location, type of watering system and the date of collection. Following proper labelling, all collected samples were promptly transported to the laboratory within a maximum of 2 h.

### 2.2 Laboratory Examination of Water Samples

#### 2.2.1 Chemical Examination

All water sample analyses were conducted in the laboratory of the Veterinary Hygiene and Management Department, Faculty of Veterinary Medicine, Cairo University, following the procedures recommended by Clesceri et al. (1998). The pH values of the water samples were determined using an electrometric pH meter (pHep HI 98107, Italy). TDS were measured employing a waterproof EC/TDS/NaCl %/°C meter (HI 9835, Italy). Total hardness was estimated using the ‘EDTA titrimetric method’. Chloride (Cl) levels were determined through the ‘argentometric method’. Nitrate (NO_3_^−^) concentrations were assessed using the ‘ultraviolet spectrophotometric screening method’. Sulphate (SO_4_^2−^) levels were determined using ‘the gravimetric methods with the drying of residues’ (Clesceri et al. 1998).

#### 2.2.2 Microbiological Examination

TCC was assessed using the pour plate method, while the TCFC was determined using the multiple tube fermentation technique, following the procedures outlined in Clesceri et al. (1998).

#### 2.2.3 Dip‐Slides

Incubation and evaluation procedures were conducted following the manufacturer's manual and technical sheet guidelines, as outlined in ISO 4833:2003 (2003).

### 2.3 Statistical and Data Analysis

The data were analysed using Statistical Package for Social Sciences software, version 25.0 (SPSS Inc., Chicago, IL). Initially, all questionnaire information was converted into variables. Data normality was assessed using the Kolmogorov–Smirnov test. Descriptive and inferential statistics for non‐parametric data, including the Wilcoxon signed‐rank test, Kruskal–Wallis *H* test, Spearman rank correlation and linear regression, were employed to present the findings. The impact of various farm risk factors on PIs was evaluated using Kruskal–Wallis *H* tests, which provided mean ranks, Kruskal–Wallis *H* values and eta‐squared measures of association. The effect size was determined using Cohen's *d* and eta‐squared value. Significance was established at a *p* < 0.05, following the guidelines of Campbell and Swinscow (2011).

## 3 Results

The survey included a total of 132 farms across Egypt, categorized by region as follows: 46 farms in the West Delta (17 in Behira and 29 along the Alexandria desert road), 12 farms in the Middle Delta (6 in Menoufia and 6 in Gharbia), 52 farms in the East Delta (6 in Kaluobia, 7 in Sharkia, 6 in Dakahlia and 33 along the Ismailia desert road) and 22 farms in Upper Egypt (16 in Fayoum and 6 in Beni‐Suef and Minya combined).

As detailed in Table 1, the surveyed cattle operations varied in size and type. Small operations (less than 100 cattle) accounted for 2.3%, medium operations (100–500 cattle) made up 40.9%, and large operations (more than 500 cattle) comprised 56.8%. These classifications follow the guidelines established by Blau et al. (2005). Although the farms were selected based on convenience, they provide a representative sample of Egypt's cattle population across diverse regions and operational scales.

**TABLE 1:** Percentage (%) of herds classified by both size and operation type.

	Percentage (%) by herd size^a^			
Operation type	Small	Medium	Large	Total
Dairy	0.76	20.45	24.24	45.45
Beef	0.76	18.94	25.76	45.45
Mixed	0.76	1.52	6.82	9.09
Total	2.27	40.91	56.82	100

^a^ Small < 100 head; medium, 100–500 head; large, > 500 head.

The questionnaire survey collected 132 responses, one from each farm, detailing descriptive items and risk factors across the surveyed operations. Key descriptive statistics for these items are summarized in Table 2.

**TABLE 2:** Percentage (%) of risk factors profile and descriptive items of the survey farms.

Variable	%
**Farm records type**	
Computerized	66
Handwritten	34
**Animal ID type**	
Electronic ID	17
Collars	11
Ear tag	71
Branding	1.5
**Cattle breeds**	
Holstein‐Friesian	94
Simmental	2.1
Brown‐Swiss	0.7
Crossbreed	2.1
Baladi	0.7
**Housing type**	
Loose/free stalls	65
Cubicle/free Stalls	0.7
Open tie‐stall	16
Closed tie‐stall	18
**Bedding type**	
Sand	66
Soil	0.8
Straw	31
Artificial mats	2.3
**Ventilation type**	
Open	81
Closed	19
**Cooling system**	
No cooling	45
Sprinkler	42
Foggers	13
Cooling pads	0.8
**Physical contact**	
No contact	71
Sheep	14
Beef	6.8
Buffalo	4.5
Goat	3.8
Donkey	3
Dog	1.5
Horse	0.8
Poultry	1.5
Camel	0.8
**Waste handling**	
Composting	24
Picket dam	36
Left on pasture	29
Landfill	9.8
Alley scraper	0.8
Manure pack	0.8
**Water source**	
Underground	69
Tap	24
Surface	6.8
**Drinkers’ type**	
Troughs	96
Automatic cups	3.8
**Drinkers’ lining**	
Ceramic	22
Cement	70
Stainless steel	3.8
Galvanized steel	1.5
Aluminum	2.3
Plastic	0.8
**Water pipes type**	
Metal	51
Plastic	49
**Water tanks type**	
Concrete	46
Fibreglass	16
Galvanized steel	34
Plastic	4.5
**Hoof dip disinfectant**	
Absent	51
CuSo4	29
Formalin	16
CuSo4+ZnSo4	3.8
Formalin+CuSo4	0.8
**Disinfectant change**	
Each 200 cow	28
Each 250 cow	12
Each 500 cow	60
**Teat dip disinfectant**	
Absent	9.7
Iodophors	85
Sodium hypochlorite	4.2
QACs	1.4

PIs for beef cattle, including DMI, FBW, FCR, IW, WG, FP and IA, were recorded, with their frequencies presented in Table 3. For dairy farms, PIs such as FE, DMY and DMI were documented, as shown in Table 4.

**TABLE 3:** Frequency three quartiles (Q1, Q2 [median], Q3) of beef performance indicators—Total dry matter intake per season (DMI), final body weight (FBW), feed conversion rate (FCR), initial weight (IW), weight gain (WG), fattening period (FP), initial age (IA)—in the survey beef farms.

Percentiles	DMI (kg)	FBW (kg)	FCR	IW (kg)	WG (kg)	FP (days)	IA (days)
Q1	1587.9	460.25	6.13	240	212	255	190
Q2	1914.5	490	7.95	250	240	255	195
Q3	2117.8	508.75	9.88	255	260	260	195

*Note*: Percentiles equal frequency quartiles (quartiles are the alternative to the arithmetic mean in non‐normally distributed data) and Q2 is the median.

**TABLE 4:** Frequency three quartiles (Q1, Q2 [median], Q3) of dairy performance indicators: Fed efficiency (FE), daily milk yield (DMY), dry matter intake (DMI) in the survey dairy farms.

Percentiles	FE	DMY (liter)	DMI (kg)
Q1	1.2	15.25	12.85
Q2	1.5	27	18.24
Q3	1.7	35.75	20.25

Laboratory analysis of water samples from farm drinkers revealed a broad spectrum of chemical and microbial results. While some parameters met the cattle WQ standards established by CCME (1993), others exceeded permissible limits, as summarized in Table 5. The distribution of values for each WQ parameter, including quartiles (Q1, median [Q2] and Q3), is detailed in Table 6.

**TABLE 5:** Percentage (%) of farms classified according to different drinkers' water chemical and microbial quality parameters (WQ) that are within permissible limits (Within PL) and out of permissible limit (Out PL).

WQ	pH	TDS	Hardness	Chloride	Nitrate	Sulphate	TCC	TCFC
Within PL	21.2	69.7	29.5	51.5	84.1	90.2	13.6	13.6
Out PL	78.8	30.3	70.5	48.5	15.9	9.8	86.3	86.3

**TABLE 6:** Frequency three quartiles (Q1, Q2 [median], Q3) of the water physiochemical and microbial quality parameters in the survey farms.

Percentiles	pH	TDS	Hardness	Chloride	Nitrate	Sulphate	TCC (winter)	TCC (summer)	TCFC (winter)	TCFC (summer)
Q1	8.1	305	285	150	2	66	3.7 × 10^4^	5.9 × 10^4^	3.8 × 10^3^	6.1 × 10^3^
Q2	8.4	680	472	240	4	100	30.5 × 10^5^	55 × 10^5^	2.7 × 10^5^	4.3 × 10^5^
Q3	8.8	1472.5	698	448	8	141.5	32 × 10^6^	76 × 10^6^	5.3 × 10^5^	9.7 × 10^5^

Spearman rank correlation analysis revealed statistically significant positive correlations (*p* < 0.05) between various chemical and microbial parameters. Notably, TDS showed strong positive correlations with hardness (*ρ* = 0.77), chloride (*ρ* = 0.89), nitrate (*ρ* = 0.32) and sulphate (*ρ* = 0.78). In addition, a significant positive correlation (*ρ* = 0.84) was observed between TCC and TCFC.

To evaluate seasonal effects, the Wilcoxon signed‐rank test was used to compare winter and summer microbial water parameters. The analysis identified significant differences, with mean ranks of 65.5, a *Z*‐value of 9.89 and an effect size (Cohen's *d*) of 0.86, highlighting higher microbial counts during summer.

**FIGURE 1:**
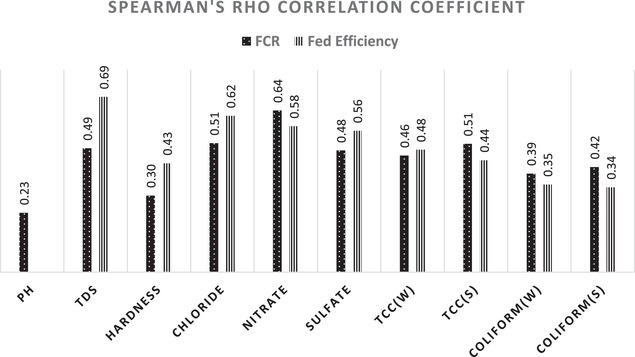
Spearman's rho correlation coefficients between water quality parameters and cattle performance indicators (FCR for beef and FE for dairy). Only parameters with a *p* < 0.05 are included in the analysis.

Furthermore, Spearman correlation analysis showed significant relationships (*p* < 0.05) between all analysed WQ parameters—except pH—and FCR, a critical PI in beef farms. Similarly, strong correlations were found between these parameters and FE, the primary PI in dairy farms. Figure 1 provides a visual representation of the Spearman correlation coefficients for beef FCR and dairy FE with significant WQ parameters.

**FIGURE 2:**
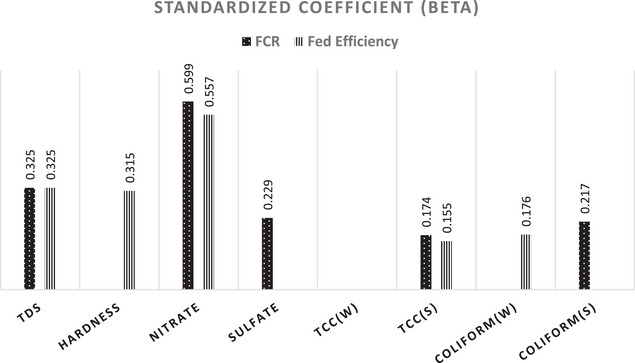
Beta values from linear regression analysis showing the predictive strength of water quality parameters for beef FCR and dairy FE. Only significant predictors (*p* < 0.05) are displayed.

Linear regression with the stepwise method was used to determine the most detrimental WQ parameters and their influence on PIs. For beef FCR, nitrate emerged as the most significant predictor, followed by TDS, sulphate, summer TCFC and summer TCC, with standardized *β* values of 0.599, 0.325, 0.229, 0.217 and 0.174, respectively. The model explained 66.8% of the variance (*R* ^2^ = 0.668). Similarly, for dairy FE, nitrate was the top predictor, followed by TDS, hardness, winter TCFC and summer TCC, with *β* values of 0.557, 0.325, 0.315, 0.176 and 0.155, respectively. This model accounted for 76.3% of the variance (*R* ^2^ = 0.763). These findings are illustrated in Figure 2.

**FIGURE 3:**
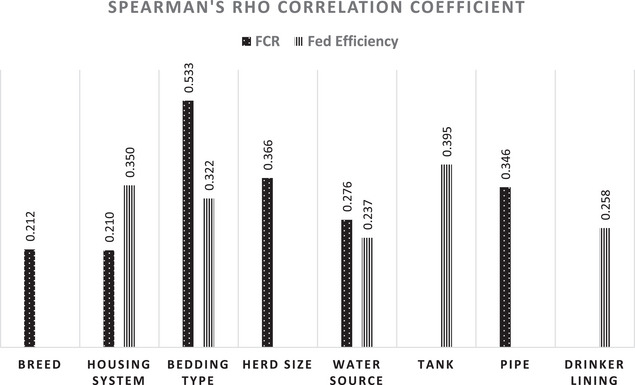
Spearman's rho correlation coefficients between farm risk factors and cattle performance indicators (FCR for beef and FE for dairy). Only risk factors with a *p* < 0.05 are included in the analysis.

Spearman rank correlation analysis also revealed significant relationships (*p* < 0.05) between beef FCR and several farm risk factors, including animal breed (*ρ* = 0.212), housing system (*ρ* = 0.210), bedding type (*ρ* = 0.533), herd size (*ρ *= 0.366), water source (*ρ* = 0.276) and water pipes type (*ρ* = 0.346). Similarly, dairy FE showed significant correlations with housing system (*ρ* = 0.350), bedding type (*ρ* = 0.322), water tank type (*ρ* = 0.237) and drinker lining type (*ρ* = 0.395). These relationships are visually represented in Figure 3.

Housing system type significantly influenced beef FCR. Mean ranks for open tie‐stall, loose/free stalls, and closed tie‐stall systems were 42.86, 35.69 and 31.5, respectively, with an eta‐squared value of 0.039. Similarly, the housing system type affected dairy FE, with mean ranks of 8.5, 35.74, 59.5 and 50 for open tie‐stall, loose/free stalls, closed tie‐stall and cubicle/free stalls, respectively. The associated eta‐squared value for dairy FE was approximately 0.116.

Bedding type also showed a notable impact on beef FCR, with mean ranks of 28.67 for straw, 24 for soil and 47.32 for sand and an eta‐squared value of 0.255. For dairy FE, bedding types yielded mean ranks of 26.5 for straw, 52.69 for soil, 31.74 for sand and 50 for artificial mats, with an eta‐squared value of 0.169.

The type of water source significantly influenced beef FCR. Mean ranks were 43.17 for underground water, 19.74 for tap water and 51.57 for surface water, with an eta‐squared value of 0.276. For dairy FE, water source also played a role, with mean ranks of 33.55, 52.29 and 8.5 for underground, tap and surface water, respectively, and an eta‐squared value of 0.156.

Water tank type affected dairy FE, with mean ranks of 51 for plastic tanks, 45.25 for galvanized steel, 55.25 for fibreglass and 30.02 for concrete tanks, and an eta‐squared value of 0.182. Similarly, drinker lining type influenced dairy FE, with mean ranks of 42.8 for stainless steel, 51 for plastic, 41.96 for ceramic and 31.54 for cement, and an eta‐squared value of 0.079.

Water pipe type significantly impacted beef FCR, with mean ranks of 40.34 for plastic pipes and 23.06 for metal pipes, and an eta‐squared value of 0.091.

Herd size showed a clear effect on beef FCR, with mean ranks of 42.67 for large herds, 27.85 for medium herds and 20.5 for small herds, and an eta‐squared value of 0.136. Cattle breed also influenced beef FCR, with mean ranks of 37.42 for Holstein Friesian, 15.5 for Crossbreed and 15 for Baladi breeds, and an eta‐squared value of 0.042.

## 4 Discussion

Drinking WQ plays a critical role in livestock health and production but is highly susceptible to variations due to water sources and contamination, which can significantly impact cattle performance (Kamal et al. 2023). This study examined the relationship between drinking WQ and key PIs in beef and dairy cattle, aiming to identify potential health and production challenges posed by substandard WQ.

### 4.1 WQ Exceedances and Their Implications

A notable proportion of surveyed farms (13%–86.3%) exhibited WQ parameters exceeding the permissible limits set by the CCME (1993) guidelines for livestock, including pH, TDS, hardness, chloride, nitrate, sulphate, TCC and TCFC. These exceedances highlight widespread WQ issues that may affect cattle health and performance. Elevated TDS levels, for example, often indicate mineral accumulation from water sources, while high microbial counts suggest inadequate hygienic practices, such as inconsistent flushing of water troughs or untreated water supplies. Addressing these exceedances is essential to optimize cattle productivity and mitigate health risks.

### 4.2 Statistical Relationships Between WQ and PIs

The statistical analysis revealed significant correlations between WQ parameters and beef FCR as well as dairy FE. For beef FCR, parameters such as TDS, hardness, chloride, nitrate, sulphate and microbial counts (TCC and TCFC) were moderately correlated, with weaker correlations observed for pH (Figure 1). Similarly, dairy FE was strongly correlated with these parameters except for pH, underscoring the consistent role of WQ in influencing both production metrics.

### 4.3 Key Predictors of PIs

Linear regression analysis identified nitrate as the most influential predictor of both beef FCR and dairy FE, with *β* values of 0.599 and 0.557, respectively (Figure 2). This finding aligns with previous research showing that nitrate levels in water can significantly affect dairy herd indicators (Raisbeck 2020; Kamal, Kaoud, et al. 2024; Kamal, Khalf, et al. 2024). In the rumen, nitrate converts to nitrite, posing toxicity risks unique to ruminants (Weichenthal et al. 1963). Chronic exposure to high nitrate levels is associated with reduced cattle production, vitamin A deficiencies (Al‐Qudah et al. 2009) and impaired immune and thyroid functions (Tyagi et al. 2022). These findings suggest that nitrate management should be a priority in improving cattle performance.

TDS emerged as the second most significant predictor of PIs, with *β* values of 0.325 for both beef FCR and dairy FE. High TDS levels, often indicative of poor WQ, have been linked to reduced feed and water intake, growth and production (Zimpel et al. 2018; Kamal et al. 2025). However, some studies suggest that TDS effects may vary based on other interacting WQ parameters, such as chloride and sulphate concentrations (Patience 1989). This highlights the need for a holistic approach to WQ management. Some researchers have suggested that high TDS levels may not necessarily pose a significant problem and may not impact animal health and production (Phillips et al. 2015).

Water hardness also influenced dairy FE (*β* = 0.315), potentially through its impact on water intake, milk production and bone mineralization (CCME 1993). While high hardness levels can predispose cattle to various health issues, some studies report negligible effects, possibly due to adaptive mechanisms (Looper and Waldner 2002). These contradictory findings warrant further investigation into the long‐term effects of water hardness on livestock.

Sulphate, with a *β* value of 0.229 for dairy FE, is another critical factor. High sulphate levels can cause laxative effects, reduce water and feed intake and interact with essential minerals like copper and selenium, leading to deficiencies (McKenzie et al. 2009; Beede 2012). However, ruminal adaptation may mitigate some of these adverse effects over time (Sharma and Kumar 2020). Understanding the threshold at which sulphate levels become detrimental is vital for effective water management.

Microbial WQ, particularly TCC during summer, emerged as a significant predictor of PIs. For beef FCR, TCC had a *β* value of 0.174, while for dairy FE, it was 0.155. These values highlight the stronger influence of TCC compared to TCFC, whose *β* values were 0.217 and 0.176 for summer and winter, respectively. These results align with previous studies (Samaha et al. 2012; Elfadl et al. 2015; Mohammed 2016), emphasizing the critical role of maintaining clean and hygienic water in cattle operations. This seasonal difference, supported by a high effect size (Cohen's *d* = 0.86), aligns with previous findings that microbial growth intensifies during warmer months due to favourable conditions (West 2003). Mitigating seasonal microbial contamination through regular water system maintenance is essential to safeguard cattle performance.

High microbial counts in water can negatively affect water palatability, reduce intake, and impair production, immunity and overall performance. These impacts can lead to significant health challenges and productivity losses in cattle. However, not all studies agree on the extent of these effects; some have reported minimal impacts of microbial contamination on cattle performance (Jensen and Vestergaard 2021). This variability underscores the need for further research to delineate the conditions under which microbial contamination becomes detrimental.

### 4.4 Correlations Among WQ Parameters

Statistical correlation analysis revealed significant positive relationships among water chemical parameters. TDS correlated strongly with chloride (*ρ* = 0.89), sulphate (*ρ* = 0.78) and hardness (*ρ* = 0.77), reflecting the interdependence of these variables (Patience 1989; Looper and Waldner 2002). Similarly, microbial parameters showed strong correlations, with TCC positively associated with TCFC (*ρ* = 0.84) (Sharma and Bhattacharya 2017; Mulhern et al. 2021; El‐Sharawya et al. 2024). These correlations underscore the importance of comprehensive WQ assessments to identify potential risks.

### 4.5 Farm Risk Factors and Their Impact on PIs

To assess the influence of various farm risk factors on PIs, statistical analysis was conducted based on data from the study questionnaire (Table 2). Housing system type showed a weak correlation with FCR (*ρ* = 0.210) and a moderate correlation with dairy FE (*ρ* = 0.210). Open tie‐stall housing had the greatest effect on beef FCR, with a mean rank of 42.86, while closed tie‐stall housing was most favourable for dairy FE, achieving a mean rank of 56.5, consistent with findings by Samer (2011). This may be attributed to environmental stressors and space limitations in different housing designs, which can influence feed intake and metabolic efficiency. Open tie‐stall housing, with greater exposure to environmental fluctuations, could increase energy expenditure for thermoregulation, negatively impacting beef FCR. Conversely, controlled environments minimize heat stress and improve feed utilization in closed tie‐stall housing. Bedding type also influenced performance, with moderate correlations observed for beef FCR (*ρ* = 0.533) and dairy FE (*ρ* = 0.322). Sand bedding had the highest impact on beef FCR (mean rank: 47.32), whereas soil bedding showed the strongest effect on dairy FE (mean rank: 52.69), in agreement with studies by Frétin et al. (2018) and Fregonesi et al. (2007). These results highlight bedding's role in comfort, hygiene and feed efficiency.

Water source type was moderately correlated with beef FCR (*ρ* = 0.276) and weakly correlated with dairy FE (*ρ* = 0.237). Surface water demonstrated the most positive effect on beef FCR (mean rank: 51.57), while tap water had the highest effect on dairy FE (mean rank: 52.29), aligning with Abdelhafiz et al. (2021). Surface water likely provides a more natural mineral balance, improving beef FCR, while tap water may offer more consistent quality, supporting better dairy FE. This highlights the importance of WQ in optimizing cattle performance. Water tank type also showed a moderate correlation with dairy FE (*ρ* = 0.395), with fibreglass tanks contributing the highest impact (mean rank: 55.25), as reported by Zhang et al. (2022). Tank material affects WQ and cattle hydration. Fibreglass tanks likely maintain better water cleanliness and temperature control, promoting improved dairy FE. Similarly, the drinker lining type demonstrated a moderate correlation with dairy FE (*ρ* = 0.258), with plastic linings yielding the greatest effect (mean rank: 51), in line with findings by Bédard et al. (2016). Material affects water access and cleanliness. Plastic linings likely offer smoother surfaces, reducing contamination and improving dairy FE.

Water pipe type exhibited a moderate correlation with beef FCR (*ρ* = 0.346), with plastic pipes proving more effective (mean rank: 40.34) than metal pipes, corroborating research by Liu et al. (2016). Pipe material impacts water flow and quality. Plastic pipes are likely more effective in maintaining water hygiene and temperature, improving feed conversion. Herd size also moderately correlated with beef FCR (*ρ* = 0.366), with large herds achieving the highest effect (mean rank: 42.67), consistent with Robbins et al. (2016). Larger herds may benefit from economies of scale, improving overall feed efficiency. Larger herds are likely to have more consistent management practices, leading to better performance. In addition, cattle breed showed a weak correlation with beef FCR (*ρ* = 0.212), with Holstein Friesian cattle outperforming other breeds (mean rank: 37.42), as observed by Faid‐Allah et al. (2018). Breed‐specific factors, such as metabolism and growth rate, influence feed efficiency. Holstein Friesian cattle likely perform better due to their superior growth potential.

When evaluating effect sizes using eta‐squared, water source had the largest influence on beef FCR (*η* ^2^ = 0.276), while water tank type had the most significant impact on dairy FE (*η* ^2^ = 0.182). These findings underscore the multifaceted nature of cattle performance, where WQ, infrastructure and management practices interact to shape outcomes.

## 5 Conclusions

The quality of drinking water significantly impacts beef and dairy PIs, with notable correlations among various water chemical parameters, including TDS, hardness, chloride, sulphate and nitrate levels. In addition, there are correlations in the levels of total coliform count and total coliform count between each other. Seasonal variations in water microbial counts were observed, and other risk factors and hygienic standards were also found to influence certain PIs.

Further research is needed to investigate the effects of WQ on calf health and performance. In addition, more studies are required to explore waterborne microbes, their resistant strains and biofilm formation in water sources used for cattle consumption. These areas of investigation can provide valuable insights into improving livestock health and productivity in livestock settings.

## Author Contributions

All authors contributed to the study's conception and design. Conceptualization: Mohammed A. Kamal, Mahmoud A. Khalaf, Zakia A. M. Ahmed, Jakeen ELjakee, Hossam Mahmoud, Rashed A. Alhotan, Elsayed Osman Hussein, Branislav Galik, and Ahmed Ali Saleh. Data curation: Mohammed A. Kamal, Mahmoud A. Khalaf, Zakia A. M. Ahmed, Jakeen ELjakee, Hossam Mahmoud, Rashed A. Alhotan, Elsayed Osman Hussein, Branislav Galik, and Ahmed Ali Saleh. Formal analysis: Mohammed A. Kamal, Mahmoud A. Khalaf, Zakia A. M. Ahmed, Jakeen ELjakee, Hossam Mahmoud, Rashed A. Alhotan, Elsayed Osman Hussein, Branislav Galik, and Ahmed Ali Saleh. Funding acquisition: Mohammed A. Kamal, Mahmoud A. Khalaf, Zakia A. M. Ahmed, Jakeen ELjakee, Hossam Mahmoud, Rashed A. Alhotan, Elsayed Osman Hussein, Branislav Galik, and Ahmed Ali Saleh. Investigation: Mohammed A. Kamal, Mahmoud A. Khalaf, Zakia A. M. Ahmed, Jakeen ELjakee, Hossam Mahmoud, Rashed A. Alhotan, Elsayed Osman Hussein, Branislav Galik, and Ahmed Ali Saleh. Methodology: Mohammed A. Kamal, Mahmoud A. Khalaf, Zakia A. M. Ahmed, Jakeen ELjakee, Hossam Mahmoud, Rashed A. Alhotan, Elsayed Osman Hussein, Branislav Galik, and Ahmed Ali Saleh. Project administration: Mohammed A. Kamal, Mahmoud A. Khalaf, Zakia A. M. Ahmed, Jakeen ELjakee, Hossam Mahmoud, Rashed A. Alhotan, Elsayed Osman Hussein, Branislav Galik, and Ahmed Ali Saleh. Resources: Mohammed A. Kamal, Mahmoud A. Khalaf, Zakia A. M. Ahmed, Jakeen ELjakee, Hossam Mahmoud, Rashed A. Alhotan, Elsayed Osman Hussein, Branislav Galik, and Ahmed Ali Saleh. Software: Mohammed A. Kamal and Ahmed Ali Saleh. Supervision: Mohammed A. Kamal, Mahmoud A. Khalaf, Zakia A. M. Ahmed, and Ahmed Ali Saleh. Validation: Mohammed A. Kamal, Mahmoud A. Khalaf, Zakia A. M. Ahmed, Jakeen ELjakee, Hossam Mahmoud, Rashed A. Alhotan, Elsayed Osman Hussein, Branislav Galik, and Ahmed Ali Saleh. Visualization: Mohammed A. Kamal, Mahmoud A. Khalaf, Zakia A. M. Ahmed, Jakeen ELjakee, Hossam Mahmoud, Rashed A. Alhotan, Elsayed Osman Hussein, Branislav Galik, and Ahmed Ali Saleh. Writing–original draft: Mohammed A. Kamal and Ahmed Ali Saleh. Writing–review and editing: Mohammed A. Kamal and Ahmed Ali Saleh.

## Ethics Statement

The Cairo University Institutional Animal Care and Use Committee (CU‐IACUC), Veterinary Medical and Agricultural Sciences Sector, granted ethical approval for this work under the code “VET CU 09092024900.” The “Guide for the Care and Use of Laboratory Animals,” issued by the Institute of Laboratory Animal Research, was followed by the Faculty of Veterinary Medicine at Cairo University for the procedures involving all the animals in this study (Washington, DC, USA). The ARRIVE 2.0 guidelines are adhered to in all animal procedures carried out in this investigation.

## Consent

The authors have nothing to report.

## Conflicts of Interest

The authors declare no conflicts of interest.

### Peer Review

The peer review history for this article is available at https://publons.com/publon/10.1002/vms3.70261

## Supporting information



Supporting Information

## Data Availability

Data is contained within the article.

## References

[vms370261-bib-0001] Abdelhafiz, M. A. , A. A. Elnazer , E.‐M. M. Seleem , et al. 2021. “Chemical and Bacterial Quality Monitoring of the Nile River Water and Associated Health Risks in Qena–Sohag Sector, Egypt.” Environmental Geochemistry and Health 43: 4089–4104. 33772385 10.1007/s10653-021-00893-3

[vms370261-bib-0002] Al‐Qudah, K. M. , L. M. Rousan , and K. I. Ereifej . 2009. “Nitrate/Nitrite Poisoning in Dairy Cattle Associated With Consumption of Forages Irrigated With Municipally Treated Wastewater.” Toxicological & Environmental Chemistry 91: 163–170.

[vms370261-bib-0003] Alves, J. N. , G. G. L. Araújo , S. G. Neto , et al. 2017. “Effect of Increasing Concentrations of Total Dissolved Salts in Drinking Water on Digestion, Performance and Water Balance in Heifers.” Journal of Agricultural Science 155: 847–856. 10.1017/S0021859617000120.

[vms370261-bib-0004] Bédard, E. , M. Prévost , and E. Déziel . 2016. “ *Pseudomonas aeruginosa* in Premise Plumbing of Large Buildings.” MicrobiologyOpen 5: 937–956. 27353357 10.1002/mbo3.391 PMC5221438

[vms370261-bib-0005] Beede, D. K. 2012. “What Will Our Ruminants Drink?” Animal Frontiers 2: 36–43.

[vms370261-bib-0006] Blau, D. M. , B. J. McCluskey , S. R. Ladely , et al. 2005. “Salmonella in Dairy Operations in the United States: Prevalence and Antimicrobial Drug Susceptibility.” Journal of Food Protection 68: 696–702. 15830658 10.4315/0362-028x-68.4.696

[vms370261-bib-0007] Campbell, M. J. , and T. D. V. Swinscow . 2011. Statistics at Square One. John Wiley & Sons.

[vms370261-bib-0008] CCME . 1993. “Protocols for Deriving Water Quality Guidelines for the Protection of Agricultural Water Uses.” CCREM 1987. Canadian Council of Ministers of the Environment. 10.1006/rtph.1994.1074 7724832

[vms370261-bib-0009] Clesceri, L. S. , A. E. Greenberg , and A. D. Eaton . 1998. Standard Methods for the Examination of Water and Wastewater. American Public Health Association.

[vms370261-bib-0010] El Emam, I. A. , and I. M. El Jalii . 2010. “Bacterial Contamination of Drinking Water in Selected Dairy Farms in Sudan.” Scientific Journal of King Faisal University 11: 153–160.

[vms370261-bib-0011] Elfadl, E. A. A. , A. M. Fardos , and H. A. A. Radwan . 2015. “Quantitative Methods to Determine Factors Affecting Productivity and Profitability of Beef Fattening Enterprises in Egypt Department of Animal Wealth Development.” Global Veterinaria 14: 77–82. 10.5829/idosi.gv.2015.14.01.9273.

[vms370261-bib-0012] El‐Sharawya, M. E. , Y. S. Hussein , A. S. A. El‐Enin , et al. 2024. “Using Lactoferrin and N‐acetylcysteine to Augment the Growth Rate and Hemato‐Biochemical Parameters of Egyptian Baladi Goats Kids.” Cogent Food & Agriculture 10, no. 1: 2351041. 10.1080/23311932.2024.2351041.

[vms370261-bib-0013] Faid‐Allah, E. , E. Ghoneim , A. R. Elbetagy , and M. El‐Dabour . 2018. “Genetic Diversity and Structure of Native Egyptian Cattle Populations and French‐Egyptian Cross via DNA‐Microsatellite.” Indonesian Journal of Animal and Veterinary Sciences (Jurnal Ilmu Ternak Dan Veteriner) 23, no. 1: 1–10. 10.14334/jitv.v23i1.1647.

[vms370261-bib-0014] Fregonesi, J. A. , D. M. Veira , M. A. G. Von Keyserlingk , and D. M. Weary . 2007. “Effects of Bedding Quality on Lying Behavior of Dairy Cows.” Journal of Dairy Science 90: 5468–5472. 18024737 10.3168/jds.2007-0494

[vms370261-bib-0015] Frétin, M. , B. Martin , E. Rifa , et al. 2018. “Bacterial Community Assembly From Cow Teat Skin to Ripened Cheeses Is Influenced by Grazing Systems.” Scientific Reports 8: 200. 29317671 10.1038/s41598-017-18447-y PMC5760519

[vms370261-bib-0016] ISO 4833:2003 . 2003. Microbiology of Food and Animal Feeding Stuffs—Horizontal Method for the Enumeration of Microorganisms—Colony Count Technique at 30°C . ISO.

[vms370261-bib-0017] ISO 18593:2004 . 2004. Microbiology of Food and Animal Feeding Stuffs‐ Horizontal Method for Sampling Techniques From Surfaces Using Contact Plates and Swabs . ISO.

[vms370261-bib-0018] Jensen, M. B. , and M. Vestergaard . 2021. “Invited Review: Freedom From Thirst—Do Dairy Cows and Calves Have Sufficient Access to Drinking Water?” Journal of Dairy Science 104: 11368–11385. 34389150 10.3168/jds.2021-20487

[vms370261-bib-0019] Kamal, M. A. , M. A. Khalaf , Z. A. M. Ahmed , et al. 2023. “Effect of Water Organic Load and Total Ammonia Nitrogen on Broilers' Humoral Immune Response Against Newcastle Disease Virus Vaccination in Egypt.” International Journal of Veterinary Science 12: 107–113. 10.47278/journal.ijvs/2022.164.

[vms370261-bib-0020] Kamal, M. A. , Z. A. M. Ahmed , M. A. Khalaf , and J. K. El‐Jakee . 2019. “Effect of Water Quality Parameters on some Health and Reproductive Indicators in Cattle Farms Associated Emerged Epidemics in Egypt.” International Journal of Veterinary Sciences 8: 275–282.

[vms370261-bib-0021] Kamal, M. A. M. , H. A. Kaoud , H. M. Salem , et al. 2024. “Revolutionizing Poultry Hygiene: Advanced Electrostatic and Cold Fog Disinfection Strategies Combat *Mycoplasma gallisepticum* in Hatching Eggs.” Poultry Science 103: 103732. 10.1016/j.psj.2024.103732. PMC11259725 38925079

[vms370261-bib-0022] Kamal, M. A. M. , M. A. Khalf , Z. A. Mohamed , et al. 2024. “Seasonal Variations Alter Drinking Water Quality at Different Points in the Water Distribution Systems of Cattle Farms in Egypt.” Egyptian Journal of Veterinary Science 56, no. 4: 757–768.

[vms370261-bib-0023] Kamal, M. A. M. , H. M. Salem , R. A. Alhotan , et al. 2025. “Unraveling Antimicrobial Resistance Dynamics in *Mycoplasma gallisepticum*: Insights into Antibiotic and Disinfectant Interactions.” Veterinary Medicine and Science 11: e70181. 10.1002/vms3.70181. 39792050 PMC11720739

[vms370261-bib-0024] Liu, S. , C. Gunawan , N. Barraud , S. A. Rice , E. J. Harry , and R. Amal . 2016. “Understanding, Monitoring, and Controlling Biofilm Growth in Drinking Water Distribution Systems.” Environmental Science & Technology 50: 8954–8976. 27479445 10.1021/acs.est.6b00835

[vms370261-bib-0025] Looper, M. L. , and D. N. Waldner . 2002. Water for Dairy Cattle. Guide D‐107. New Mexico State University, Cooperative Extension Service.

[vms370261-bib-0026] Manzanilla‐Pech, C. I. V. , R. F. Veerkamp , R. J. Tempelman , et al. 2016. “Corrigendum to “Genetic Parameters Between Feed‐Intake‐Related Traits and Conformation in 2 Separate Dairy Populations—the Netherlands and United States” (*J. Dairy Sci*. 99:443–457).” Journal of Dairy Science 99: 4095. 26547641 10.3168/jds.2015-9727

[vms370261-bib-0027] McKenzie, R. A. , A. M. Carmichael , M. L. Schibrowski , S. A. Duigan , J. A. Gibson , and J. D. Taylor . 2009. “Sulfur‐Associated Polioencephalomalacia in Cattle Grazing Plants in the Family Brassicaceae.” Australian Veterinary Journal 87: 27–32. 19178473 10.1111/j.1751-0813.2008.00387.x

[vms370261-bib-0028] Mohammed, A. N. 2016. “Field Study on Evaluation of the Efficacy and Usability of Two Disinfectants for Drinking Water Treatment at Small Cattle Breeders and Dairy Cattle Farms.” Environmental Monitoring and Assessment 188: 151. 10.1007/s10661-016-5147-0. 26861741

[vms370261-bib-0029] Mulhern, R. , M. Stallard , H. Zanib , J. Stewart , E. Sozzi , and J. M. Gibson . 2021. “Are Carbon Water Filters Safe for Private Wells? Evaluating the Occurrence of Microbial Indicator Organisms in Private Well Water Treated by Point‐of‐Use Activated Carbon Block Filters.” International Journal of Hygiene and Environmental Health 238: 113852. 34627100 10.1016/j.ijheh.2021.113852

[vms370261-bib-0030] Patience, J. F. 1989. “Water Quality and Quantity: Importance in Animal and Poultry Production.” In Biotechnology in the Feed Industry. Proceedings of Alltech's Fifth Annual Symposium, edited by T. P. Lyons , 121–138. Alltech Technical Publications.

[vms370261-bib-0031] Phillips, C. J. C. , M. O. Mohamed , and P. C. Chiy . 2015. “Effects of Duration of Salt Supplementation of Sheep on Rumen Metabolism and the Accumulation of Elements.” Animal Production Science 55: 603–610.

[vms370261-bib-0032] Raisbeck, M. F. 2020. “Water Quality for Grazing Livestock I.” Veterinary Clinics: Food Animal Practice 36: 547–579. 32943304 10.1016/j.cvfa.2020.08.014

[vms370261-bib-0033] Robbins, J. A. , M. A. G. Von Keyserlingk , D. Fraser , and D. M. Weary . 2016. “Invited Review: Farm Size and Animal Welfare.” Journal of Animal Science 94: 5439–5455. 28046157 10.2527/jas.2016-0805

[vms370261-bib-0034] Saleh, A. A. , M. M. Soliman , M. F. Yousef , et al. 2023. “Effects of Herbal Supplements on Milk Production Quality and Specific Blood Parameters in Heat‐Stressed Early Lactating Cows.” Frontiers in Veterinary Science 10: 1180539. 10.3389/fvets.2023.1180539. 37332736 PMC10274320

[vms370261-bib-0035] Samaha, H. A. , Y. N. Haggag , M. A. Nossair , et al. 2012. “Epidemiological Survey on Environmental Bacterial Pathogen Causing Mastitis in Cattle.” Alexandria Journal of Veterinary Sciences 37, no. 1: 41–47.

[vms370261-bib-0036] Samer, M. 2011. “Effect of Cowshed Design and Cooling Strategy on Welfare and Productivity of Dairy Cows.” Journal of Agricultural Science and Technology 1: 848–857.

[vms370261-bib-0037] Sharma, M. K. , and M. Kumar . 2020. “Sulphate Contamination in Groundwater and Its Remediation: An Overview.” Environmental Monitoring and Assessment 192: 74. 31897853 10.1007/s10661-019-8051-6

[vms370261-bib-0038] Sharma, S. , and A. Bhattacharya . 2017. “Drinking Water Contamination and Treatment Techniques.” Applied Water Science 7: 1043–1067.

[vms370261-bib-0040] Tyagi, J. , S. Ahmad , and M. Malik . 2022. “Nitrogenous Fertilizers: Impact on Environment Sustainability, Mitigation Strategies, and Challenges.” International Journal of Environmental Science and Technology 19: 11649–11672.

[vms370261-bib-0041] West, J. W. 2003. “Effects of Heat‐Stress on Production in Dairy Cattle.” Journal of Dairy Science 86: 2131–2144. 12836950 10.3168/jds.S0022-0302(03)73803-X

[vms370261-bib-0039] Weichenthal, B. A. , L. B. Embry , R. J. Emerick , and F. W. Whetzal . 1963. “Influence of Sodium Nitrate, Vitamin A and Protein Level on Feedlot Performance and Vitamin A Status of Fattening Cattle.” Journal of Animal Science 22: 979–984.

[vms370261-bib-0042] Zhang, H. , D. Liu , L. Zhao , et al. 2022. “Review on Corrosion and Corrosion Scale Formation Upon Unlined Cast Iron Pipes in Drinking Water Distribution Systems.” Journal of Environmental Sciences 117: 173–189. 10.1016/j.jes.2022.04.024 35725069

[vms370261-bib-0043] Zimpel, R. , M. B. Poindexter , A. Vieira‐Neto , et al. 2018. “Effect of Dietary Cation‐Anion Difference on Acid‐Base Status and Dry Matter Intake in Dry Pregnant Cows.” Journal of Dairy Science 101: 8461–8475. 29970257 10.3168/jds.2018-14748

