# Supplementary material for: Effects of Drinking Water Quality Parameters on Egyptian Cattle Farm Performance Indicators
Source: Vet Med Sci. 2025 Feb 21;11(2):e70261. doi: 10.1002/vms3.70261 (PMC11843028; doi:10.1002/vms3.70261)
Supplement: Supplementary file 1 — Supporting Information [file VMS3-11-e70261-s001.pdf]

Support File. 1

The questionnaire structured by research team for the study farms survey and includes:

1. General Information.

1. Visit date
2. Farm name
3. Full address
4. Governorate
5. Operation type
6. No. of animals (dairy, heifer, calves, beef)
7. Average herd size
8. Breed

2. Data about cattle house.

**House**	Housing system type
	Ventilation system
	Cooling system
	Calf house type
**Floor**	Floor type
	Bedding type
**Feeders**	Feeders type
	Feeders lining
	Calf Feeders' type
**Disposal method**	Waste disposal
	Carcass disposal

3. Data about water system.

**Water source**	
**Water tanks type**	
**Water pipes type**	
**Drinkers**	Type
	Lining
	Water appearance
	Floor around drinkers

4. Data about disinfection and disinfectants.

**Water disinfection**	Disinfectant type
	Frequency of use and change
**Drinkers disinfection**	Disinfectant type
	Frequency of use and change
**Water tanks disinfection**	Disinfectant type
	Frequency of use and change
**Water pipes disinfection**	Disinfectant type
	Frequency of use and change
**General farm disinfection**	Disinfectant type
	Frequency of use and change
**Floor disinfection**	Disinfectant type
	Frequency of use and change
**Wheel dip disinfection**	Disinfectant type
	Frequency of use and change
**Foot dip disinfection**	Disinfectant type
	Frequency of use and change
**Hoof dip disinfection**	Disinfectant type
	Frequency of use and change
**Milk house disinfection**	General disinfectant type
	Teat dip
	Milk line
	Milk tanks
**Feeder disinfection**	Disinfectant type
	Frequency of use and change
**Calf feeder's disinfection**	Disinfectant type
	Frequency of use and change

5. Data about some health conditions

**Epidemics**	Type
	Morbidity rate
	Mortality rate
**Reproductive status in dairy**	Rate of difficult birth (Dystocia %)
	Retained placenta %
	Metritis %
	Mastitis %
	Average DO
	Average NIPC
	Average conception rate
	Infertility %
	Abortion %
	Lameness %
	Culling %

**Calf's health status**	Diarrhea %
	Respiratory Problems %
	Severe Depression %
	Sudden Death %
	Mortality %

6. Data about some performance parameters

**Water intake / head**
**Feed intake (DMI) / head**
**Average final body weight**
**Average daily milk yield**
